# Could paraprobiotics be a safer alternative to probiotics for managing cancer chemotherapy-induced gastrointestinal toxicities?

**DOI:** 10.1590/1414-431X2022e12522

**Published:** 2023-01-16

**Authors:** L.M.S. Nobre, C. Fernandes, K.G.D. Florêncio, N.M.N. Alencar, D.V.T. Wong, R.C.P. Lima-Júnior

**Affiliations:** 1 Núcleo de Pesquisa e Desenvolvimento de Medicamentos, Departamento de Fisiologia e Farmacologia, Faculdade de Medicina, Universidade Federal do Ceará, Fortaleza, CE, Brasil

**Keywords:** Diarrhea, Probiotics, Paraprobiotics, Cancer, Chemotherapy

## Abstract

Clinical oncology has shown outstanding progress improving patient survival due to the incorporation of new drugs. However, treatment success may be reduced by the emergency of dose-limiting side effects, such as intestinal mucositis and diarrhea. Mucositis and diarrhea management is symptomatic, and there is no preventive therapy. Bacterial and fungal-based compounds have been suggested as an alternative for preventing the development of diarrhea in cancer patients. Using probiotics is safe and effective in immunocompetent individuals, but concerns remain during immunosuppressive conditions. Paraprobiotics, formulations composed of non-viable microorganisms, have been proposed to overcome such limitation. The present literature review discusses current evidence regarding the possible use of paraprobiotics as an alternative to probiotics to prevent gastrointestinal toxicity of cancer chemotherapy.

## Introduction

The survival of patients undergoing chemotherapy or radiotherapy has increased in the last decades due to considerable advances in drug therapy, a better understanding of cancer pathogenesis, and increased awareness of the population's importance of early disease diagnosis (1). The evolvement of cancer treatment led to the development of personalized and more selective therapies. However, the high cost of some modern therapeutic approaches limits patient access to precision medicine in specific cancer centers, especially in developing countries, where most cancer-related deaths are concentrated (2,3).

Therapeutic regimens used worldwide are still based on non-selective cytotoxic drugs and promote unwanted side effects that compromise therapeutic efficacy and can contribute to increased mortality due to toxicities (4). Among toxicities, mucositis affects the entire gastrointestinal tract and is accompanied by pain, vomiting, and diarrhea, usually leading to treatment interruption or reduction in the chemotherapy dose intensity. Clinical management of gastrointestinal mucositis is symptomatic, and there is still no preventive or curative treatment (5).

The intestinal microbiota in healthy individuals plays an essential role in maintaining intestinal homeostasis, with protective effects on epithelial integrity. The mechanism seems to involve bacterial interaction with toll-like receptors and activation of the NF-κB signaling pathway, avoiding mucosal damage and stimulating cell repair and regeneration (6,7). Additionally, the mucus produced by the intestinal epithelial cells and the expression of intercellular junction proteins are essential components of the intestinal barrier against pathogens (8). Anticancer drugs promote dysbiosis that induces changes in intestinal permeability and inflammation. The use of bacteria-based formulations to balance the intestinal microbiota is recognized for its health benefits and can mitigate the deleterious impact of chemotherapy on the gastrointestinal tract (9).

Currently, there is a wide variety of bacteria-composed products, like probiotics, prebiotics, symbiotics, postbiotics, and paraprobiotics. Table 1 shows the definitions and potential applications of these compounds. Probiotics, composed of live organisms, exert their effects through modulation of the immune response, activation of regulatory cytokines, maintenance of the epithelial barrier integrity, and competition with pathogenic microorganisms. It is a complementary therapy that quantitatively and qualitatively alters intestinal microbiota composition (10).

**Table 1:** Probiotic, paraprobiotic, and postbiotic definitions and uses.

	Definition	Commonly used species	Application
Probiotic	Live microorganisms that confer a health benefit when consumed in adequate amounts (66).	Genus Lactobacillus: *Lactobacillus acidophilus, L. casei, L. paracasei, L. rhamnosus, L. delbrueckii subsp. bulgaricus, L. brevis, L. johnsonii, L. plantarum and L. fermentum* (67), genus Bifidobacterium: *Bifidobacterium infantis, B. adolescentis, B. animalis subsp animalis, B. animalis subsp lactis, B. bifidum, B. longum, B. breve* (68), Saccharomyces: *S. boulardii* (69,70).	Diarrhea (71,72), irritable bowel syndrome (73), constipation (74), diabetes (75), depression (76).
Paraprobiotic	Inactivated microbial cells or cell fractions that confer health benefit to the host (77).	*Lactobacillus casei* (59,60), *Lacticaseibacillus casei* (61), *Lactobacillus rhamnosus* GG (78), *Saccharomyces boulardii* (64), *Lactobacillus gasseri* (63), *Enterococcus faecalis* (79), *Lactobacillus paracasei* (80), *Lactobacillus crispatus* (81).	Intestinal mucositis (65), modulation of the microbiota and improvement of intestinal function (63), obesity (82).
Postbiotic	Soluble factors (metabolites, metabiotics, supernatants, or cell extracts) released by the lysis of bacteria or secreted by live bacteria, causing benefits to the host (57).	*Lactobacillus amylovorus* (83), *Escherichia coli* (84), *Enterococcus faecalis* (84), *Haemophilus influenzae, Streptococcus pneumoniae, Klebsiella pneumoniae ssp. pneumoniae, Klebsiella pneumoniae ssp. ozaenae, Staphylococcus aureus, Streptococcus pyogenes, Streptococcus sanguinis* and *Moraxella (Branhamella) catarrhalis* (Broncho-Vaxom^®^) (85-87).	Obesity 9^83^), atopic dermatitis (84), sinusitis (85), asthma (86), prevention of respiratory infections (87).

Probiotics have not been associated with the appearance of significant adverse effects in immunocompetent individuals. On the other hand, the safety of such compounds in immunocompromised patients should be carefully analyzed (11). Recent studies have evaluated whether bacterial viability is essential for their health benefits. The verification that these positive effects can also be achieved with dead bacteria, known as paraprobiotics, without certain risks of administering a live organism is of great importance. The present review explores the perspectives of using paraprobiotics as an alternative to probiotics to prevent the gastrointestinal toxicities of cancer chemotherapy.

## Material and Methods

The search strategy was performed using MEDLINE (via PubMed) database, where the search terms were “Paraprobiotics” OR “Heat-killed Probiotic” OR “Non-viable Probiotic” OR “Ghost Probiotic” OR “Probiotics”. We combined the terms, through the Boolean operator AND, with the MeSH descriptors “Cancer”, “Adverse Effects”, “Safety”, “Diarrhea”, and “Clinical Trial”. We selected original articles or case reports in English according to their relevance to the review topic.

## Chemotherapy-associated intestinal microbiota dysbiosis and diarrhea

Cancer treatment induces intestinal mucositis, which considerably worsens the quality of life of patients (12). Diarrhea is one of the most important and debilitating manifestations of intestinal mucositis. The frequency and occurrence of diarrhea depend on the drug used and the therapeutic regimen. For instance, the weekly regimen of irinotecan or 5-fluorouracil (5-FU) causes the highest rate of this adverse effect, with more than 10% of patients developing grade 3 or 4 diarrhea (13). The chemotherapeutic agent irinotecan is a significant aggravating factor. The FOLFIRI regimen (5-FU, leucovorin, and irinotecan) triggers diarrhea in 89% of patients. Additionally, therapy with FOLFOX (5-FU, leucovorin, and oxaliplatin) induces any diarrhea grade in 56% of patients (14). Conversely, patients exposed to other anticancer drugs, such as doxorubicin and methotrexate, also manifest some degree of gastrointestinal toxicities (15,16). Despite diarrhea and intestinal mucositis being common side effects in patients undergoing cancer therapy, the mechanisms that trigger them are not fully understood, though some advancements have been seen (17).

There are several types of cancer therapy-related diarrhea: secretory, osmotic, exudative, malabsorptive, caused by dysmotility, infectious, inflammatory, and steatorrhea (18). Clinical reports suggest that chemotherapy-induced diarrhea may develop due to changes in intestinal absorption accompanied by excessive fluid secretion, or it may be a consequence of chemotherapy's biochemical and inflammatory changes (18). Current knowledge is mainly obtained from animal models using irinotecan or 5-FU. Chemotherapy administration damages the intestine, which is accompanied by excessive mucus secretion. Irinotecan induces diarrhea due to malabsorption of water and electrolytes and increases mucin secretion (19). Several apoptotic cells in the intestinal epithelium and colon, combined with the increase in goblet cells, decrease the absorptive capacity considerably, triggering diarrhea (20).

Such toxicity is primarily associated with intestinal microbiota dysbiosis (21,22). It is not yet clear how the drugs alter gut microbiota composition. Indeed, animal models and clinical trials indicate that irinotecan, 5-FU, and radiotherapy modify the gut microbiota. After treatment with irinotecan, for example, the number of beneficial bacteria such as *Lactobacillus* spp. and *Bifidobacterium* spp. decreases, while the amount of *Staphylococcus* spp., *Clostridium* spp., and *E. coli* increases (23). Additionally, 5-FU increases anaerobic bacteria in the oral cavity and facultative anaerobes in the large intestine, culminating with mucositis development (7,24). Notably, the presence of bacteria increases in the cervical and mesenteric lymph nodes (25). Stringer et al. (23), treating mice with a single dose of irinotecan, observed an increase in *Escherichia spp.*, *Clostridium spp*., *Enterococcus spp.*, *Serratia spp.*, and *Staphylococcus spp*. in the microbiota of the stomach, intestine, and colon. In stool, there was an increase of *Proteus spp*., *Clostridium spp*., and *Peptostreptococcus spp*., along with a decrease in *Bacillus spp*., and *Bifidobacterium spp*.

The incidence of dysbiosis, though, is described for other antitumor agents. In a study of patients with non-Hodgkin's lymphoma treated with Carmustine, Etoposide, Aracytine, or Melphalan, the feces of these patients were analyzed before and after chemotherapy. Chemotherapy caused qualitative changes in the composition of the microbiota, with a reduction in bacteria of the *Firmicutes* and *Actinobacteria* phylum and an increase in the genera *Clostridium*, *Bifidobacterium*, *Citrobacter*, *Klebsiella*, *Enterococcus*, *Megasphaera*, and *Parabacteroides* (26). In another study, paclitaxel-treated mice showed reduced fecal and colonic microbiota diversity but a relative increase in the presence of the *Lactobacillus* genus (27). The relationship between unbalanced gut microbiota and chemotherapy-derived gastrointestinal toxicities strongly points toward solving dysbiosis as a strategy to prevent or treat such side effects.

## Effects of probiotics on the gastrointestinal toxicities of cancer chemotherapy

To re-establish the balance of the gut microbiota, the use of probiotics is an option. Some studies reveal that the incorporation of probiotics as a therapeutic alternative to prevent or improve the gastrointestinal symptoms of chemotherapy is an approach that shows favorable outcomes. In order to deliver good effects, probiotic use must start at least one month before the beginning of antineoplastic therapy. Among the types of therapy, probiotics have shown to be more effective in preventing the intestinal effects induced by radiotherapy than chemotherapy (28).

The Colon Dophilus^TM^ probiotic (composed of *Bifidobacterium breve*, *Bifidobacterium bifidum*, *Bifidobacterium longum*, *Lactobacillus rhamnosus*, *Lactobacillus acidophilus*, *Lactobacillus casei*, *Lactobacillus plantarum*, *Streptococcus thermophilus*, *Lactobacillus brevis*, and *Bifidobacterium infantis*) given for 12 weeks to patients with metastatic colorectal cancer treated with irinotecan reduced diarrhea severity and decreased the overall incidence of diarrhea and enterocolitis (29). Additionally, abdominal bloating was more frequently reported by patients receiving a placebo compared to the probiotic-treated group. Patients who ingested the formula also reported less use of loperamide and atropine for the symptomatic treatment of diarrhea (29). In children with acute leukemia presenting chemotherapy-related gastrointestinal symptoms, using a *Lactobacillus rhamnosus* probiotic at a concentration of 5×10^9^ was found to be safe and effective, preventing the development of diarrhea and reducing abdominal distention, constipation, nausea, and meteorism (30).

Another study followed up 52 patients with colorectal cancer who consumed probiotics containing six strains of *Lactobacillus* and *Bifidobacterium* for six months, starting one month after surgery (31). It demonstrated that despite probiotics reducing the cytokines TNF-α, IL-17A, IL-17C, IL-22, IL-10, and IL-12, there was no difference in diarrhea incidence compared with the group that received a placebo. However, using probiotics was shown to be safe for those patients (31). In another study, 150 patients treated with 5-FU using a *Lactobacillus rhamnosus* GG probiotic showed reduced diarrhea frequency and less abdominal discomfort, contributing to sustaining the chemotherapeutic dose intensity. Furthermore, the probiotic did not trigger adverse effects, and no bacteria were detected in the blood cultures of the patients (32).

Rodents administered with 5-FU present intestinal damage in a mechanism dependent on the full activation of toll-like receptor types 2 and 4 (TLR2 and TLR4) by pathogenic Gram-negative bacteria. TLR2 and TLR4, in turn, stimulate NF-κB and the release of pro-inflammatory cytokines that culminates with the manifestation of gastrointestinal symptoms such as nausea, pain, and diarrhea (23,33). Conversely, prophylactic use of *Saccharomyces boulardii* probiotic prevents gastrointestinal dysfunction, such as changes in gastric emptying, absorption, permeability, and intestinal transit (33). It also reduces neutrophil infiltration and expression of inflammatory markers (34). Similar effects were obtained with *Lactobacillus acidophilus* probiotic, whose administration started on the day of intestinal mucositis induction with 5-FU (35). The probiotic VSL#3 (a combination of *Lactobacillus plantarum*, *Lactobacillus casei*, *Lactobacillus acidophilus*, *Lactobacillus delbrueckii* subspecies *bulgaricus*, *Bifidobacterium infantis*, *Bifidobacterium longum*, *Bifidobacterium breve*, and *Streptococcus salivarius* subspecies *thermophilus*) exerts benefits when administered before and after treatment of mice with irinotecan. It reduces chemotherapy-induced diarrhea through three mechanisms: increasing proliferation of epithelial cells, reducing apoptosis, and preventing the increase in caliciform cells and mucin secretion (36). Probiotics containing *Lactobacillus acidophilus* and *Bifidobacterium animalis* subspecies *lactis* have also been shown to reduce the incidence and severity of radiotherapy-induced diarrhea and abdominal pain. Remarkably, loperamide is the only anti-diarrheic medication used for controlling chemotherapy-associated diarrhea, but with limited effectiveness (37).

## Are probiotics safe?

The quality control in the development and production of bacteria-derived products merits improvement. Better strain identification is highly desirable to avoid contaminants, like pathogens, that may compromise the safety of these compounds (38). According to a report jointly published by the World Health Organization (WHO) and the Food and Agriculture Organization of the United Nations (FAO), probiotics can be responsible for four types of side effects: systemic infections, harmful metabolic activities, gene transfer, and excessive immune stimulation in susceptible individuals (39). Most cases of probiotic-derived adverse events are associated with the genera *Lactobacillus* and *Bifidobacterium*. They include bacteremia, endocarditis, and liver abscess (Table 2).

**Table 2:** Probiotics-related side effects.

Side effects	Probiotic species	Patients	References
Bacteremia	*Lactobacillus rhamnosus*	Adult with severe ulcerative colitis treated with corticosteroids and mesalazine	(42)
	*Lactobacillus rhamnosus* GG	A child with ulcerative colitis	(43)
	*Lactobacillus rhamnosus* GG	A child with the short gut syndrome	(44)
	*Lactobacillus rhamnosus*	Patient with hepatic cirrhosis	(49)
	*Lactobacillus paracasei*	Patient with prostate cancer in remission	(50)
	*Bifidobacterium longum*	Preterm infants	(51,88)
	*Bifidobacterium breve*	Newborn with multiple abdominal organ abnormalities	(52)
	*Bifidobacterium* spp.	A child with heart disease	(53)
	*Lactobacillus acidophilus*	Immunosuppressed patient	(54)
Epidural and retropharyngeal abscess	*Lactobacillus rhamnosus*	Adult with severe ulcerative colitis	(45)
Hepatic abscess	*Lactobacillus rhamnosus*	Adult with hypertension and diabetes	(46)
Endocarditis	*Lactobacillus rhamnosus*, *Streptococcus faecalis*, and *Lactobacillus acidophilus*	Adult with mild mitral valve regurgitation	(47)
	*Lactobacillus rhamnosus*	Adult with gingival ulceration	(48)

Among the species that cause bacteremia, *Lactobacillus rhamnosus* has been one of the most prevalent (40,41). Bacteremia was reported in a patient with severe ulcerative colitis using a probiotic containing *L. rhamnosus*. The same strain was isolated in the patient's blood and had the same vancomycin resistance profile as the strain in the probiotic (41). Bacteremia (42) was also described in a patient with ulcerative colitis treated with corticosteroid and infliximab, who used an *L. rhamnosus* GG probiotic (43), and in a child with the short gut syndrome (44). Sometimes, patients developed epidural and retropharyngeal abscesses (45) or liver abscesses (46).

One case of endocarditis was associated with probiotics (composed of *Lactobacillus rhamnosus*, *Streptococcus faecalis*, and *Lactobacillus acidophilus*) used to balance the intestinal microbiota after amoxicillin. That patient presented a positive blood culture for *L. rhamnosus* (47). Despite being one of the most used genera as a probiotic, *Lactobacillus* is associated with several cases of endocarditis, most of them in patients who had undergone dental surgery or had some gingival damage (48). The presence of damage in other organs can also be a gateway for these microorganisms to reach the bloodstream, as in a case of liver cirrhosis (49) and in a case of a patient with prostate cancer in remission, who habitually consumed probiotics, who presented positive bacteremia for *Lactobacillus paracasei* after colonoscopy (50).

There are also some reported cases of bacteremia and sepsis development regarding treatment with Bifidobacterium, including premature newborn babies with necrotizing enterocolitis (51) and a neonate with multiple abdominal organ abnormalities (52). Additionally, bacteremia was found in a child with heart disease who used probiotics to prevent antibiotic-induced diarrhea (53).

Bacteremia was also found in an immunocompromised individual diagnosed with Acquired Immune Deficiency Syndrome and Hodgkin's disease. The patient, who had completed the first cycle of chemotherapy, was using a probiotic based on *L. acidophilus*, and cultures obtained from the peripheral blood and the previous catheter site were positive for *Lactobacillus spp.* (54).

These cases support the hypothesis that viable bacteria, although safe for healthy individuals, can be dangerous in immunocompromised subjects, including cancer patients undergoing myelosuppressive chemotherapy. Careful individual assessment is highly demanded since these microorganisms can potentially cause life-threatening infections. Thus, paraprobiotics, composed of inactivated microorganisms, could be considered for immunosuppressed patients.

## Biological activities of paraprobiotics

The term paraprobiotic was first used by Taverniti and Guglielmetti (55) to define non-viable microbial cells or cell fractions that confer human or animal benefits when administered in adequate amounts. Paraprobiotics can be produced by inactivation with heat, high pressure, radiation, and sonication (56). It contrasts with soluble factors originating from bacterial lysis or secreted by live bacteria, called postbiotics (57). These compounds have proven immunomodulatory, antioxidant, and anti-inflammatory activity in *in vitro* and *in vivo* assays, but the mechanisms are not fully elucidated (58).

Although the benefits of using products containing bacteria are independent of cell viability, the mechanisms by which inactivated strains promote their positive effects are still a matter of debate. In a murine model of toxoplasmosis, *Lactobacillus casei* paraprobiotic increases monocyte chemoattractant protein (MCP-1) production, reduces the percentage of Tregs cells, and parasite load (59). *Lactobacillus paracasei* paraprobiotic induces IgA production and the expression of IL-10, IL-21, STAT4, and Bcl-6, inducing follicular Th cells differentiation (60). Using *Lacticaseibacillus casei* in rats promotes the modulation of the microbiota by increasing the proportion of beneficial bacteria compared to harmful ones, prevents the increase in total and low-density lipoprotein cholesterol, and controls insulin resistance in rats (61). The immunomodulatory effect of *Lactobacillus casei* Zhan paraprobiotic was described on macrophages, with increased iNOS and IL-6 expression. Unlike the *L. casei* probiotic, there was increased expression of TLR2, TLR3, TLR4, and TLR9 after six hours of exposure to the non-viable bacteria (62).

In humans, the effects of paraprobiotic use are also noted, especially on the gastrointestinal tract. One hundred and eighteen healthy adults consuming *Lactobacillus gasseri* CP230 paraprobiotic daily for three weeks underwent daily questionnaires about their quality of life and stool characteristics. The subjects showed modulation of the intestinal microbiota and improvement in the number and odor of stools (63).

## Are paraprobiotics suitable for treating chemotherapy-associated diarrhea?

Despite the few reports about paraprobiotics, they are believed to produce similar effects to probiotics. *L. paracasei* paraprobiotic, when orally administered in mice, induces antigen-specific IgA production in the small intestine, serum, and lungs and increases the proportion of follicular helper T cells in Peyer's patches (60). In a model of intestinal obstruction in mice, *S. boulardii* paraprobiotic promoted immunomodulation with increased production of the anti-inflammatory cytokine IL-10 and reduced intestinal lesions (60,64). Brandão et al. (61) showed that inactivated *Lacticaseibacillus casei* bacteria modulate the intestinal microbiota of mice, increasing the proportion of beneficial bacteria (*Lachnospiraceae* and *Ruminoccocaceae*) and reducing the number of harmful species, such as *Clostridiaceae*, *Enterobacteriaceae*, and *Helicobacteriacea*. *Enterococcus faecalis* paraprobiotic administration for one week before chemotherapy reduced the intestinal damage in the ileum of irinotecan-injected mice. It also reduced the presence of neutrophils and macrophages and abolished bacteremia. The mechanism involved the maintenance of zonula occludens protein integrity (65).

Dead bacteria and non-viable fungi from *Saccharomyces boulardii* were shown to prevent bacterial translocation and increase intestinal permeability in a murine model of intestinal obstruction. Besides increasing cytokine IL-10 and IgA levels, *S. boulardii* paraprobiotic reduces intestinal mucosal lesions in these animals (64). One study further reported that the daily use of *Lactobacillus gasseri* CP230 paraprobiotic improves the quality of life of healthy individuals by modulating the intestinal microbiota, the number of bowel movements, and stool odor (63). These studies provide evidence for using paraprobiotics to manage chemo- and radiotherapy-induced diarrhea. They may be as effective as probiotics and have a better safety profile.

## Conclusions

Despite probiotics' efficacy in modulating intestinal microbiota, there is a growing concern regarding their use in immunocompromised subjects. The literature provides novel evidence supporting the indication of paraprobiotics with no loss of effectiveness compared with probiotics to control chemotherapy-associated dysbiosis. Conversely, the benefits of other bacterial strains to compose paraprobiotic-based formulations need to be validated. Producing paraprobiotics formulations is feasible and facilitates the applicability of such compounds in the clinical setting. Additionally, the potential advantages of paraprobiotics include the safety profile and reduction in the risk of systemic infection and transference of antimicrobial resistance mechanisms. Clinical trials are warranted to delineate rational therapeutic protocols based on paraprobiotics to manage the gastrointestinal manifestations of cancer treatment toxicities.
